# Angiotensin II-Induced Hypertension Is Attenuated by Overexpressing Copper/Zinc Superoxide Dismutase in the Brain Organum Vasculosum of the Lamina Terminalis

**DOI:** 10.1155/2016/3959087

**Published:** 2016-01-06

**Authors:** John P. Collister, Heather Taylor-Smith, Donna Drebes, David Nahey, Jun Tian, Matthew C. Zimmerman

**Affiliations:** ^1^Department of Veterinary and Biomedical Sciences, College of Veterinary Medicine, University of Minnesota, St. Paul, MN 55108, USA; ^2^Department of Cellular and Integrative Physiology, University of Nebraska Medical Center, Omaha, NE 68198, USA

## Abstract

Angiotensin II (AngII) can access the brain via circumventricular organs (CVOs), including the subfornical organ (SFO) and organum vasculosum of the lamina terminalis (OVLT), to modulate blood pressure. Previous studies have demonstrated a role for both the SFO and OVLT in the hypertensive response to chronic AngII, yet it is unclear which intracellular signaling pathways are involved in this response. Overexpression of copper/zinc superoxide dismutase (CuZnSOD) in the SFO has been shown to attenuate the chronic hypertensive effects of AngII. Presently, we tested the hypothesis that elevated levels of superoxide (O_2_^∙−^) in the OVLT contribute to the hypertensive effects of AngII. To facilitate overexpression of superoxide dismutase, adenoviral vectors encoding human CuZnSOD or control adenovirus (AdEmpty) were injected directly into the OVLT of rats. Following 3 days of control saline infusion, rats were intravenously infused with AngII (10 ng/kg/min) for ten days. Blood pressure increased 33 ± 8 mmHg in AdEmpty rats (*n* = 6), while rats overexpressing CuZnSOD (*n* = 8) in the OVLT demonstrated a blood pressure increase of only 18 ± 5 mmHg after 10 days of AngII infusion. These results support the hypothesis that overproduction of O_2_^∙−^ in the OVLT plays an important role in the development of chronic AngII-dependent hypertension.

## 1. Introduction

Many years ago the anterior ventral portion of the hypothalamus lining the third ventricle (AV3V) was implicated in playing a role in almost every form of experimental hypertension [1, 2]. This hypothalamic region is comprised primarily of the organum vasculosum of the lamina terminalis (OVLT), median preoptic nucleus (MnPO), and efferent fibers of the subfornical organ (SFO) [3]. Both the SFO and OVLT are known circumventricular organs (CVOs) devoid of the normal blood brain barrier and have been shown to be directly responsive to actions of angiotensin II (AngII) [4, 5]. While not a CVO, the MnPO of the lamina terminalis has both anatomical and functional connections to/from the SFO and OVLT [6–11], and its lesion or disruption from these other brain nuclei has been shown to inhibit the AngII-induced drinking response, elevation in blood pressure, and vasopressin secretion [12–16].

In recent years, our laboratory has been dissecting the individual components of this AV3V region and their role in chronic AngII-induced hypertension in order to better understand the exact involvement of these specific central nuclei. Indeed, through lesions of individual components of the AV3V, we have shown a role for each of these areas in the chronic hypertensive response to AngII. In response to a 10-day infusion of AngII, we have previously shown that rats with specific lesions of each of the SFO, MnPO, and OVLT have demonstrated an attenuated hypertensive response, implicating each of these sites as having a specific role in the chronic actions of AngII [17–22].

More recently, in an attempt to investigate the underlying signaling mechanisms in the central hypertensive response to peripherally administered AngII, we investigated the role of central superoxide (O_2_^∙−^) production during AngII-induced hypertension. Previously, it has been reported that overexpression of copper/zinc superoxide dismutase (CuZnSOD), an antioxidant enzyme that specifically scavenges O_2_^∙−^, in the SFO markedly attenuates the gradual and chronic hypertension produced by peripheral administration of AngII [23]. These results were very similar to those in which we reported an attenuation of AngII-induced hypertension in rats with lesions of the SFO [17, 18]. More recently, we tested the hypothesis that elevated O_2_^∙−^ levels in the MnPO additionally play a role in the chronic hypertensive response to AngII. Utilizing direct central injections of adenovirus encoding CuZnSOD into the MnPO, we demonstrated an attenuated hypertensive response to chronic intravenously administered AngII [24]. Collectively, these previous studies have (1) demonstrated a role for each of the SFO, MnPO, and the OVLT in the chronic hypertensive response to AngII [17–22] and (2) demonstrated a role for O_2_^∙−^-dependent signaling in mediating these effects, thus far, in the SFO and MnPO [23, 24].

In the present study, we hypothesized that elevated levels of O_2_^∙−^ in the OVLT contribute to the AngII-induced increase in blood pressure observed during peripheral infusion of AngII. To test this hypothesis, we overexpressed CuZnSOD specifically in the OVLT of rats by utilizing direct injections of adenoviral vectors encoding CuZnSOD into the OVLT. Rats were instrumented with telemetric blood pressure measuring transducers and peripherally infused with AngII for 10 days, as we have previously described [17, 19]. Our results demonstrate that the chronic hypertensive effects of AngII are reduced in rats with overexpression of CuZnSOD in the OVLT.

## 2. Materials and Methods

All experiments were approved by the University of Minnesota Institutional Animal Care and Use Committee (IACUC) and conducted according to guidelines of the National Institutes of Health. Adult male Sprague Dawley (Charles River Laboratory, Wilmington, MA, USA) rats (250–275 g) were used in all experiments. Animals were housed in an IACUC approved and monitored facility with a 12-hour day/night light cycle (lights on 7:00 AM).

### 2.1. Surgical Procedures

Rats were anesthetized with an intraperitoneal injection of ketamine (75 mg/kg) and xylazine (10 mg/kg). Rats were then placed in a stereotaxic apparatus with the head level and fixed. A dorsal midline skull incision was made and a 2 mm hole was drilled in the skull just caudal to bregma. Replication-deficient adenoviral vectors encoding human CuZnSOD (AdCuZnSOD) or control adenovirus (AdEmpty) (Viraquest Inc., North Liberty, IA) were pair matched at titers of 10^9^ pfu/mL. Using a Hamilton syringe, 50 nL of either virus was directly injected into the dorsal OVLT of rats (*n* = 8 AdCuZnSOD; *n* = 6 AdEmpty). The coordinates used for injection caudal and ventral to bregma, respectively, were as follows in mm: (+0.65, −7.95). The hole in the skull was repaired with bone wax and the skin was closed with 3-0 silk suture. After completion of surgery, all rats were given antibiotic and analgesic injections (gentamicin; 2.5 mg, I.M. and butorphanol tartrate; 0.075 mg, S.C., resp.).

After one week of recovery, all rats were instrumented with blood pressure monitoring radiotelemeter devices (model number TA11PA-C40, Data Sciences International, St. Paul, MN) and femoral venous catheters, as we previously described [17, 20]. Rats were anesthetized as described above and an abdominal incision was made to expose and clamp the abdominal aorta proximally. The distal aorta was punctured with a 19-gauge needle and the tip of the transducer catheter was introduced into the aorta and secured in place with tissue adhesive. The body of the transducer was secured to the abdominal wall and the incision was closed. All rats were given antibiotics and analgesics as described above and individually placed in metabolic cages upon recovery. Rats were started on a continuous IV isotonic saline infusion of 7 mL/24 hr and given a 0.4% NaCl diet and water* ad libitum*.

### 2.2. Experimental Protocol

Following another week of recovery, all rats entered the experimental protocol. The first three days of the protocol served as a control period during which time a continuous intravenous infusion of 0.9% sterile saline (7 mL/24** **hr) was maintained. This was followed by a 10-day infusion of AngII (10** **ng/kg/min) which was dissolved in 0.9% sterile saline and intravenously infused at a rate of 7** **mL/24** **hr. Finally, a 3-day recovery period identical to the control period (i.e., saline infusion) completed the protocol.

Measurements of food intake, water intake, and urine output were made daily. Twenty-four-hour sodium intake was calculated as dietary sodium intake (product of food intake and sodium content of the food (0.4% NaCl, 0.07** **mmol/g) plus infused sodium (1** **mmol/day)). Urinary sodium was measured using a NOVA-5+ ion specific electrode (Biomedical, Waltham, MA, USA). Daily urinary sodium excretion was calculated as the product of urinary sodium content and daily urine volume. Total water intake was calculated as water ingested plus 7 mL from the daily IV infusion. Balance measurements were calculated as the difference between total input and excretion.

### 2.3. Immunofluorescent Detection of CuZnSOD

Following the measurement of hemodynamic parameters, rats were euthanized and perfused with 4.0% paraformaldehyde, and brains were removed. Coronal brain sections were processed for CuZnSOD protein immunoreactivity, as previously described [23]. Briefly, brain sections were incubated overnight at 4°C with human CuZnSOD antibody (1 : 500; sheep anti-human CuZnSOD; The Binding Site, Birmingham, UK) in 2% normal horse serum and 0.3% Triton followed by incubation with donkey anti-sheep AlexaFluor 488 secondary antibody (1 : 200; Invitrogen, Molecular Probes, Carlsbad, CA). CuZnSOD immunofluorescence in the OVLT was imaged with confocal microscopy (Zeiss 510 Meta Confocal Microscope, Carl Zeiss Microscopy GmbH, Jena, Germany). Image J analysis software was used to quantify fluorescence intensity in OVLT brain sections from rats injected with AdEmpty or AdCuZnSOD. Fluorescence intensity in brain sections from AdCuZnSOD-injected is reported as fold-change versus intensity in sections from AdEmpty-injected rats.

### 2.4. Statistical Analysis

Data are reported as mean ± SE. Student's *t*-test was used to compare two groups while one- or two-way ANOVA was used for multiple comparisons combined with a Student-Newman-Keuls post hoc analysis. Differences were considered significant at *P* < 0.05.

## 3. Results

### 3.1. Adenovirus-Mediated Overexpression of CuZnSOD in the OVLT

**Figure 1:**
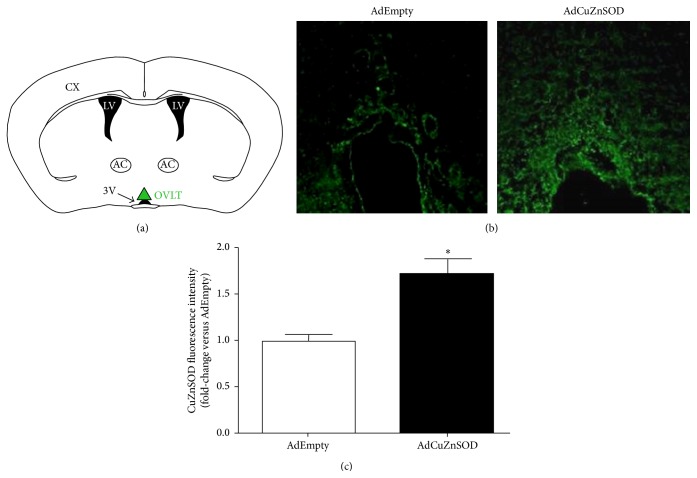
Schematic of coronal brain section showing location of OVLT (a). Representative confocal microscopy immunofluorescence images demonstrating CuZnSOD expression (green fluorescence) in the OVLT from an AdEmpty- or AdCuZnSOD-injected rat (b). Fluorescence intensity in the OVLT was quantified with Image J analysis software and is reported as fold-change in AdCuZnSOD rats (*n* = 8) versus AdEmpty rats (*n* = 6) (c). ^*∗*^ *P* < 0.05 versus AdEmpty-injected rats.

Immunofluorescence confocal microscopy was used to confirm overexpression of CuZnSOD in the OVLT in brain sections from rats that received an injection of AdCuZnSOD directly into the OVLT. Approximately 5 weeks after direct injection of either AdCuZnSOD or AdEmpty, levels of CuZnSOD were significantly elevated (1.7-fold increase, *P* < 0.05; Figure 1(c)) in the OVLT of rats receiving the AdCuZnSOD injection compared to those of rats receiving direct injection of AdEmpty. Representative confocal microscopy images showing CuZnSOD immunoreactivity are presented in Figure 1(b). It should be noted regarding the study population that all AdCuZnSOD-injected rats included in the final hemodynamic analyses (described below) were confirmed to have robust CuZnSOD expression in and confined to the OVLT relative to the low fluorescence detected in the OVLT of AdEmpty-treated rats.

### 3.2. CuZnSOD Overexpression in the OVLT Attenuates AngII-Induced Hypertension

**Figure 2:**
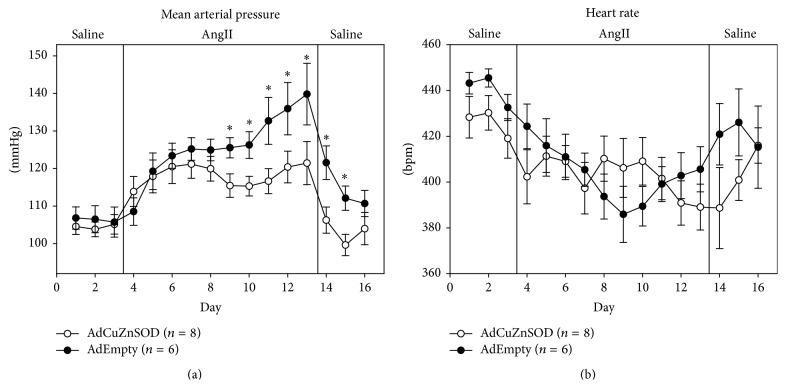
Summary data showing average 24-hour mean arterial pressure (a) and heart rate (b) recorded during saline infusion (3 days), AngII infusion (10 ng/kg/min) for 10 days, and recovery saline infusion (3 days) in rats that were OVLT injected with AdCuZnSOD (*n* = 8) or AdEmpty (*n* = 6). ^*∗*^ *P* < 0.05 versus AdCuZnSOD-injected rats.

Average baseline mean arterial pressure (MAP) was not different between AdCuZnSOD- (105 ± 2 mmHg) and AdEmpty-injected (106 ± 3 mmHg) rats during the control saline infusion period (Figure 2(a)). However, during the 10-day AngII infusion period, MAP was significantly reduced (on days 6–10 of AngII and on days 1 and 2 of the recovery period) in AdCuZnSOD-treated rats compared to MAP of AdEmpty-injected rats (Figure 2(a)). Specifically, MAP reached 121 ± 6 mmHg in AdCuZnSOD rats by day 10 of AngII infusion but increased to 140 ± 8 mmHg in AdEmpty rats. Average heart rate (HR) (Figure 2(b)) was not significantly different between the two groups during the control saline infusion period (AdCuZnSOD: 424 ± 9, AdEmpty: 440 ± 4 beats/min). In addition, while all rats were observed to have generally lower HR during AngII infusion, there was no statistical significance between the two groups.

### 3.3. CuZnSOD Overexpression in the OVLT Does Not Affect Sodium and Water Balance in AngII-Infused Hypertensive Rats

**Figure 3:**
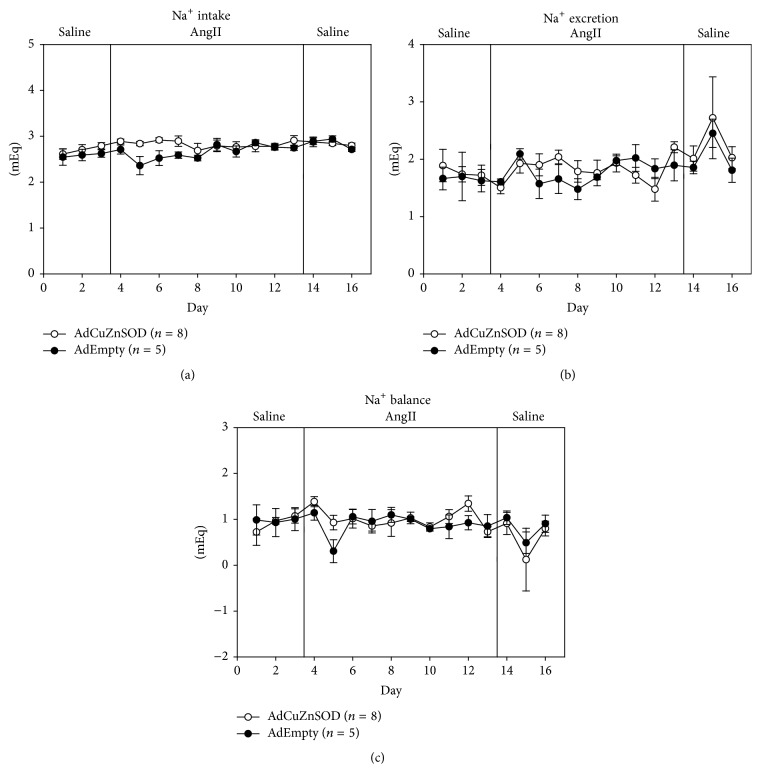
Summary data showing average 24-hour sodium intake (a), sodium output (b), and sodium balance (c) during control saline infusion and subsequent AngII infusion (10 ng/kg/min) for 10 days in rats that were OVLT injected with AdCuZnSOD (*n* = 8) or AdEmpty (*n* = 5).

**Figure 4:**
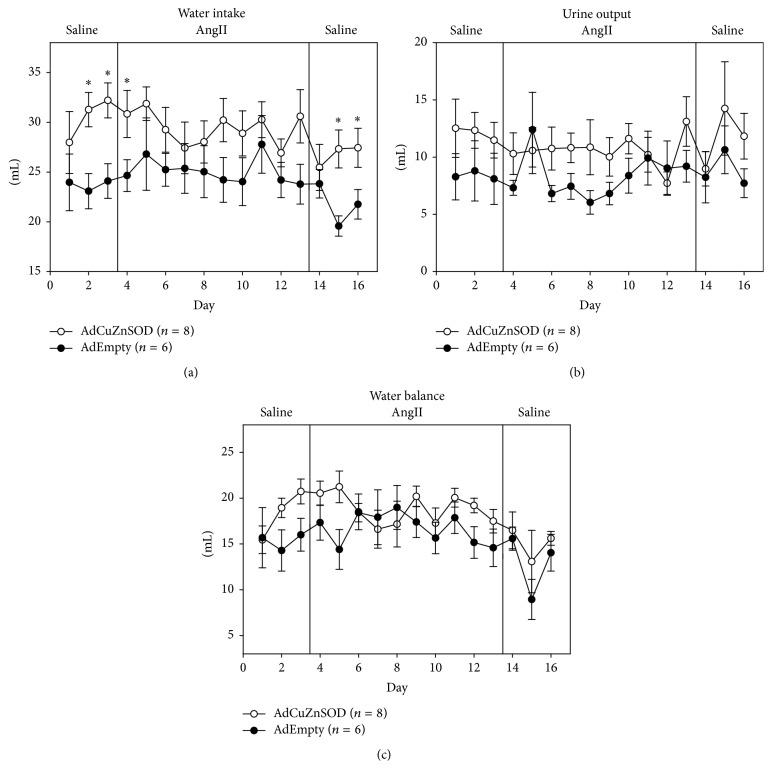
Summary data showing average 24-hour water intake (a), urine output (b), and water balance (c) during control saline infusion followed by AngII infusion (10 days; 10 ng/kg/min) and recovery saline infusion (3 days) in rats that were OVLT injected with AdCuZnSOD (*n* = 8) or AdEmpty (*n* = 6). ^*∗*^ *P* < 0.05 versus AdEmpty-injected rats.

To determine if overexpression of CuZnSOD in the OVLT affects body fluid homeostasis during AngII-induced hypertension, sodium intake, sodium excretion, and sodium balance (Figure 3), as well as water intake, urine output, and water balance (Figure 4), were measured throughout the protocol. Regarding sodium intake, sodium excretion, and sodium balance, no differences were observed between groups throughout the protocol. Regarding water intake, AdCuZnSOD-treated rats showed a significant increase on days 2 and 3 of saline infusion, day 1 of AngII treatment, and the last 2 recovery days (Figure 4(a)) and tended to have increased water intake during the protocol. However, this was offset by a slightly increased urine output throughout the protocol (Figure 4(b)). Thus, no change in overall water balance (Figure 4(c)) was observed between the groups. Collectively, overexpression of CuZnSOD in the OVLT did not alter sodium and water balance during the protocol compared to AdEmpty-injected control rats.

## 4. Discussion

Much research has been conducted examining the AngII model of hypertension and its neurogenic component(s) [25, 26]. However, we still do not have a complete understanding of the integrative pathways and central nuclei involved in the hypertensive response to peripherally administered AngII. Our laboratory has characterized and extensively used a modest dose of AngII and normal salt diet in rats to produce a reproducible and chronic hypertension model that is gradual and progressive in onset similar to human hypertension [17, 19, 20]. It does not appear to be associated with or dependent on the angiotensin converting enzyme 2 (ACE2) and angiotensin (1–7) (Ang(1–7)) axis [27], and despite known renal actions of AngII given at higher doses, we have repeatedly reported no chronic changes in sodium or water balance using this model of hypertension [17, 19, 21]. Rather, it appears that there is a significant central nervous system signaling component via CVOs associated with the chronic hypertension in this model [17–22]. By utilizing lesion studies, our laboratory has previously demonstrated that both the SFO and OVLT, as well as one of their downstream integration sites, the MnPO, are each independently necessary for the full hypertensive response to elevated AngII [17–22].

More recently, attention has also been directed toward fully understanding the intracellular signaling pathways involved in the chronic hypertensive response to AngII. In the current study, we report that overexpression of CuZnSOD, an intracellular superoxide scavenging enzyme, in the OVLT significantly attenuates the increase in MAP induced by chronic, peripheral infusion of AngII. Control rats that received an injection of an empty adenovirus into the OVLT responded to AngII with a 33 ± 8 mmHg rise in arterial pressure after 10 days of exogenous AngII, while rats overexpressing CuZnSOD in the OVLT showed a markedly attenuated response of only 18 ± 5 mmHg over the same 10 days of AngII treatment. These results support the hypothesis that overproduction of O_2_^∙−^ in the OVLT significantly contributes to the full hypertensive response to AngII.

The SFO has been one of the more extensively studied CVOs and has been widely reported as having an important role in the dipsogenic and hypertensive effects of circulating AngII [28–32]. We have previously demonstrated that the SFO is necessary to achieve the full hypertensive response to chronic peripheral AngII infusion in studies utilizing electrolytic lesion of this CVO [17, 18]. Furthermore, it has been shown that this effect is mediated, at least in part, through increased O_2_^∙−^ signaling in the SFO [23]. Thus, the SFO has clearly been given much deserved attention as having a major role in the chronic AngII-induced hypertension model. In addition to the SFO, the OVLT is a hypothalamic sensory CVO of significance and part of the originally described anterior ventral 3rd ventricle (AV3V). Along with the OVLT, this area contains the ventral part of the MnPO and the periventricular tissue surrounding the 3rd ventricle [3] and was originally characterized for its role in the mechanisms of hypertension, as lesion of this area prevents many forms of experimental hypertension including the AngII-dependent model [1, 2]. Therefore, we recently attempted to dissect the individual role of the OVLT itself in chronic AngII-dependent hypertension by discrete lesion of the OVLT followed by administration of AngII. In those studies, we observed that specific lesion of the OVLT markedly attenuated the effects of a 10-day infusion of AngII in rats [21]. In the current study, similar to what has been studied in the SFO, we aimed to further elucidate the mechanisms of this response by testing the hypothesis that increased levels of O_2_^∙−^ contribute to the long-term hypertensive effects of AngII. To do this, an adenovirus encoding CuZnSOD was injected directly into the OVLT to selectively overexpress this O_2_^∙−^ scavenging enzyme in this brain region involved in cardiovascular control. The results of the study demonstrate a strikingly similar inhibition of the rise in arterial pressure during 10 days of intravenous AngII infusion to what we have previously reported in SFO or OVLT lesioned rats [17, 21]. Furthermore, these results suggest that not only is O_2_^∙−^ playing a role in the signaling mechanism at the SFO during AngII-induced hypertension [23], but also it has at least an equally important role in the OVLT in this model of hypertension.

The present study suggests a definitive role of O_2_^∙−^ in the OVLT as an intracellular signaling mechanism in chronic AngII-induced hypertension. While these results are similar to the attenuation of the hypertensive effects of AngII that was previously reported in mice with overexpression of CuZnSOD in the SFO [23], there are some notable differences. The above-mentioned study conducted by Zimmerman et al. used a mouse model in which AdCuZnSOD was injected intracerebroventricularly (ICV), and AngII was delivered subcutaneously via osmotic minipump at a dose of 600 ng/kg/min over a period of 16 days [23]. In the current study, we used direct injection of AdCuZnSOD into the OVLT and a continuous IV infusion of AngII (10 ng/kg/min) for 10 days. In both studies, MAP gradually increased in control animals, an effect that was significantly attenuated in AdCuZnSOD-treated animals throughout the course of AngII treatment. In the above-mentioned AdCuZnSOD SFO mouse study, MAP rose to approximately 150–160 mmHg, and this effect was attenuated after 11 days of AngII infusion in mice overexpressing SOD in the SFO [23] compared to the present study, in which we report a peak MAP of 140 mmHg in control animals after 10 days of AngII treatment that was attenuated after 6 days of AngII at a level of approximately 120 mmHg in AdCuZnSOD rats. These differences are probably attributable to a number of factors including the choice of animal model, as well as the dose and route of AngII administration. Nevertheless, both studies demonstrate a chronic hypertension during AngII treatment that was significantly attenuated only after several days in animals treated with central AdCuZnSOD, equally implicating O_2_^∙−^ signaling in the OVLT as well as in the SFO as a mechanism mediating chronic AngII-induced hypertension. Another difference between previous studies and the current study is that AdCuZnSOD was directly injected into the OVLT in the present study. In contrast, Zimmerman et al. performed ICV injections of adenovirus and thus targeted the SFO nonspecifically. Nonetheless, they observed CuZnSOD overexpression predominantly in the SFO and thus concluded that increased scavenging of O_2_^∙−^ in the SFO attenuated AngII-induced hypertension [23]. Through lesion studies, our laboratory has much previous experience with specifically targeting the OVLT [21, 22] and was therefore able to utilize direct injections into the OVLT in the present study in order to specifically target this important cardiovascular control nucleus.

Downstream of the OVLT, following activation by AngII, the MnPO receives reciprocal inputs from not only the OVLT but also the SFO and is therefore believed to form part of the sympathoexcitatory pathway [10, 11, 33, 34]. In an attempt to further clarify the role of the MnPO in the development of AngII-induced hypertension, our laboratory reported similar decreases in the long-term hypertensive response to AngII in rats with either total electrolytic or chemical ablation of the MnPO [19, 20]. Building from these previous observations, we sought to shed further light on the role of O_2_^∙−^ as an intracellular signaling molecule in, specifically, the MnPO, using the same model of AngII-induced hypertension [24]. The results from that study again demonstrated a very similar attenuation of the typical development of hypertension during 10 days of exogenous AngII to what has been reported in the SFO [23] and to our current OVLT data presented herein. Collectively, these studies indicate that O_2_^∙−^-dependent signaling in the SFO, MnPO, and OVLT mediates, at least in part, the hypertensive response to elevated levels of circulating AngII.

While the observations in the MAP response were similar in the present OVLT study to those in the study using overexpression of CuZnSOD in the MnPO [24], there are some apparent differences. Overexpression of CuZnSOD in the MnPO caused an attenuated MAP response to AngII that began after only 2 days of AngII infusion and lasted throughout day 10, whereas the attenuated response to elevated AngII in rats receiving injections into the OVLT in the present study began on day 6 of AngII treatment. Furthermore, the maximal attenuation of MAP was greater in rats with overexpression of CuZnSOD in the MnPO, as MAP in those rats only increased 6 mmHg [24]. These differences are perhaps not surprising considering that if the MnPO is indeed receiving AngII-mediated signaling inputs from both the SFO and OVLT, one would expect a more dramatic attenuation in the MAP response to AngII when the signaling pathway in this integration site for more than one CVO is disrupted compared to blocking signaling mechanisms in either CVO alone. Lastly, of notable interest is that the attenuation of the MAP response in OVLT lesioned animals treated with 10 days of AngII was also much greater than the attenuated response of rats with overexpression of CuZnSOD in the OVLT. We previously reported that rats with lesions of the OVLT responded with a maximal attenuated response of only approximately 25% the rise in MAP compared to sham lesioned rats during a 10-day infusion of AngII [21]. This suggests that while O_2_^∙−^ in the OVLT is playing an important role in the intracellular signaling cascade mediating the rise in blood pressure during AngII infusion, it is not the only mechanism functioning during this model of hypertension.

In conclusion, our current results support a notable role of elevated O_2_^∙−^ in the OVLT in mediating the chronic hypertensive effects of AngII. Furthermore, we have now clearly established that O_2_^∙−^ signaling in both the SFO and OVLT, as well as the downstream MnPO, is all independently important in mediating the chronic hypertensive response to AngII. Some arguable questions remaining are as follows: which CVO is the primary site driving this response, or are they equally important, and if so are they redundant or even compensatory in their function? For example, when this pathway is blocked in one CVO, is it possible that we are not observing the maximal response due to some compensatory function or redundancy in CVO signaling, and therefore we have possibly observed the maximal attenuated response to AngII in our studies involving the MnPO? Taken together, our current and previous data support further investigations into new therapies and technologies for delivering antioxidant treatments to these definitive central nuclei in the treatment of hypertension, specifically targeting reductions in or prevention of increased O_2_^∙−^ levels.
